# Supplementary material for: Evaluation of a Novel Synthetic Peptide Derived from Cytolytic Mycotoxin Candidalysin
Source: Toxins (Basel). 2022 Oct 11;14(10):696. doi: 10.3390/toxins14100696 (PMC9610734; doi:10.3390/toxins14100696)
Supplement: Supplementary file 1 [file toxins-14-00696-s001.zip › toxins-1882042-supplementary.pdf]

toxins

MDPI

Supplementary Materials: Evaluation of a Novel Synthetic Peptide Derived from Cytolytic Mycotoxin Candidalysin

Pedro Henrique de Oliveira Cardoso, Ana Paula de Araújo Boleti, Patrícia Souza e Silva, Lincoln Takashi Hota Mukoyama, Alexya Sandim Guindo, Luiz Filipe Ramalho Nunes de Moraes, Caio Fernando Ramalho de Oliveira, Maria Ligia Rodrigues Macedo, Cristiano Marcelo Espínola Carvalho, Alinne Pereira de Castro and Ludovico Migliolo

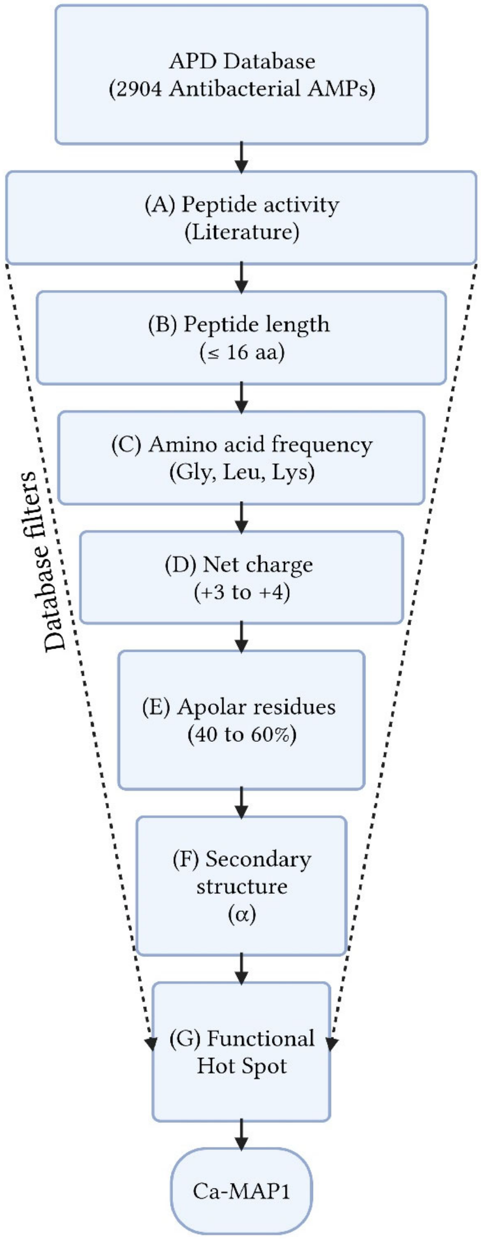

Figure S1. Database filtering technology schematic: (A) Candidalysin activity, (B) Determination of peptide length, (C) Amino acids frequency, (D) Net charge, (E) Hydrophobic content, (F) Structure type (F) Functional hot spot. The designed peptide is called Ca-MAP1.

Table S1. Strategy for the selection of peptide based on physical chemical parameters of candidalysin. Legend: <H> = Hydrophobicity in the Eisenberg scale; <μH> = Hydrophobic moment.

Sequence	Length	Net charge	Nonpolar residues (%)	<H>	<μH>
SIIGIIMGILGNIPQVIQIIMSIVKAFKGNKR	32	+4	56.25	0.679	0.408
SIIGIIMGILGNIPQV----------------	16	0	62.50	0.926	0.526
----------------IQIIMSIVKAFKGNKR	16	+4	50.00	0.432	0.409
-----------------QIIMSIVKAFKGNKR	15	+4	46.67	0.341	0.446
------------------IIMSIVKAFKGNKR	14	+4	50.00	0.381	0.463
-------------------IMSIVKAFKGNKR	13	+4	46.15	0.272	0.439
--------------------MSIVKAFKGNKR	12	+4	41.67	0.144	0.350
---------------------SIVKAFKGNKR	11	+4	36.36	0.045	0.472
----------------------IVKAFKGNKR	10	+4	40.00	0.054	0.518
-----------------------VKAFKGNKR	9	+4	33.33	-0.140	0.381
----------------IQIIMSIVKAFKGNK-	15	+3	53.33	0.528	0.382
----------------IQIIMSIVKAFKGN--	14	+2	57.14	0.636	0.464
----------------IQIIMSIVKAFKG---	13	+2	61.54	0.732	0.521
----------------IQIIMSIVKAFK----	12	+2	66.67	0.792	0.565
----------------IQIIMSIVKAF-----	11	+1	72.73	0.955	0.607
----------------IQIIMSIVKA------	10	+1	70.00	0.871	0.491
----------------MSIVKAFKG-------	9	+2	55.56	0.481	0.477
-----------------QIIMSIVKAFKGNK-	14	+3	50.00	0.437	0.410
------------------IIMSIVKAFKGN--	12	+2	58.33	0.611	0.507
-------------------IMSIVKAFKG---	10	+2	60.00	0.613	0.593

Table S2. Antibiogram assay against Gram-negative and -positive bacteria. (+) resistant at the concentrations tested.

**Microorganism**	Amoxicilin	Imipenem	Eritromicin	Ampicilin
**Gram-negative**	**>350.3 (μM)**	**>427.6 (μM)**	**>174.4 (μM)**	**>366.3 (μM)**
Acinetobacter baumannii (clinical isolated)	+	+	+	+
Escherichia coli (clinical isolated)	+	+	+	+
Escherichia coli (KPC)	+	+	+	+
Klebsiella pneumoniae (ATCC)	+	+	+	+
Klebsiella pneumoniae (KPC)	+	+	+	+
Pseudomonas aeruginosa (ATCC)	+	+	+	+
**Gram-positive**				
Staphylococcus aureus (clinical isolated)	+	+	+	+
